# Interference of blood pressure control within 24 hours in acute ischemic stroke: systematic review protocol

**DOI:** 10.1186/cc14693

**Published:** 2015-09-28

**Authors:** Arnaldo A da Silva, Alvaro N Atallah, Gisele S Silva, Gustavo José M Porfírio

**Affiliations:** 1 Universidade Federal de São Paulo, Vila Clementino, São Paulo, SP, Brazil

## 

Stroke is the third most common cause of death in most industrialized countries, with an estimated global mortality of 4.7 million yearly. A stroke occurs every 53 seconds in North America and by 2002 was projected to become the fourth leading burden of disease worldwide. Stroke killed 283,000 people in 2000 and accounted for about one in every 14 deaths in the United States. Each year, about 700,000 people suffer a new or recurrent stroke. It is the major cause of serious, long-term disability, with more than 1,100,000 American adults reporting functional limitations resulting from stroke.

Review of the evidence on how acute variation in blood pressure (BP) during the first 24 hours of acute ischemic stroke can influence outcome, considering interesting preliminary evidence that without intervening medications may be superior to some use of drugs in modifying an acute rise in BP, and suggesting that the blood pressure decline spontaneously without administration of medication may also have an influence on the acquired disabilities.

We searched the Cochrane Central Register of Controlled Trials (CENTRAL) (The Cochrane Library 2013, Issue 12), MEDLINE (1954-July 2013), EMBASE (1980-July 2013), CINAHL (1982-July 2013), Database of Research in Stroke (DORIS) (2008-2013), Latin American and Caribbean Health Sciences Literature (LILACS) (1982-December 2013) and reference lists of articles. We contacted researchers in the field. We did a grey literature search for articles published until July 2013. We also searched Dissertation Abstracts International via Dissertation Express, and the metaRegister of Controlled Trials. In an effort to identify further published, unpublished and ongoing trials, we searched ongoing trials registers and SCOPUS.

Inclusion criteria: 1, age 18-75 years; 2, clinical signs consistent with the diagnosis of ischemic stroke; 3, treatment onset within 3-9 hours after stroke onset; 4, no prior neurologic event that would obscure the interpretation of the signal and current presenting neurologic deficits (modified Rankin scale = 1); 5, National Institutes of Health Stroke Scale (NIHSS) score >4 and at least moderate limb weakness; 6, MRI screening to be started within 7.5 hours after stroke onset; 7, perfusion abnormality of >2 cm in diameter involving hemispheric gray matter; 8, perfusion/diffusion mismatch of 20; 9, magnetic resonance angiography shows TICI grade 0 or 1. Exclusion criteria: 1, prestroke score on the modified Rankin Scale >2 or on the Barthel Index.

Review authors will work independently to assess risk of bias using criteria described in the Cochrane Handbook for Systematic Reviews of Interventions (Higgins 2011) to assess trial quality. This set of criteria is based on evidence of associations between overestimate of effect and high risk of bias of the article such as sequence generation, allocation concealment, blinding, incomplete outcome data and selective reporting. If the raters disagreed, the final rating was made by consensus, with the involvement of another member of the review group. Where inadequate details of randomization and other characteristics of trials were provided, authors of the studies were contacted in order to obtain further information. Nonconcurrence in quality assessment was reported, but if disputes arose as to in which category a trial was to be allocated, again, resolution was made by discussion. The level of risk of bias was noted in both the text of the review and in the "Summary of findings tables".

Primary outcomes: death or dependency at the end of scheduled follow-up. Dependency is defined as being severely dependent on others in activities of daily living, or being significantly disabled; this corresponds to a Barthel Index score or a modified Rankin Scale grade 3-6 at 3-month follow-up.

Secondary outcomes: 1, standardized nondisease-specific instrument for describing and valuating health-related quality of life; EQ-5D (EuroQol) questionnaire. 2, NIHSS measure of neurologic deficit; the Barthel Index measure of activities of daily living; the Modified Rankin Scale measure of the degree of disability or dependence in daily activities for 90 days follow-up. 3, average time of hospital discharge. 4, time to discharge from the neuro ICU or neuro critical care unit. 5, assessment of systolic and diastolic blood pressure control. 6, causality assessment of adverse events following blood pressure reduction within 24 hours of acute ischemic stroke.
